# Evidence That p-Cresol and IL-6 Are Adsorbed by the HFR Cartridge: Towards a New Strategy to Decrease Systemic Inflammation in Dialyzed Patients?

**DOI:** 10.1371/journal.pone.0095811

**Published:** 2014-04-22

**Authors:** Eleonora Riccio, Mauro Cataldi, Maristella Minco, Gennaro Argentino, Roberta Russo, Stefania Brancaccio, Andrea Memoli, Lucia Grumetto, Loredana Postiglione, Bruna Guida, Bruno Memoli

**Affiliations:** 1 Department of Public Health, Section of Nephrology, Federico II University of Naples, Naples, Italy; 2 Department of Neuroscience, Reproductive and Odontostomatologic Sciences, Section of Pharmacology, Federico II University of Naples, Naples, Italy; 3 Department of Pharmaceutical and Toxicological Chemistry, Federico II University of Naples, Naples, Italy; 4 Department of Cellular and Molecular Biology and Pathology, Federico II University of Naples, Naples, Italy; 5 Department of Clinical Medicine and Surgery, Federico II University of Naples, Naples, Italy; University of Sao Paulo Medical School, Brazil

## Abstract

**Introduction:**

Hemodialysis (HD) and hemodiafiltration clear only with a low efficiency the plasma from interleukin-6 and p-cresol, two protein-bound uremic toxins associated with high cardiovascular risk in end stage renal disease. HFR Supra is a double-chamber hemodiafiltration system in which the ultrafiltrate returns to the patient after its regeneration through a resin cartridge that binds hydrophobic and protein-bound solutes. In the present study, we evaluated whether the HFR cartridge can also bind total p-cresol and IL-6 and remove them from the ultrafiltrate.

**Methods:**

We compared the levels of IL-6 and p-cresol in ultrafiltrate samples collected at the inlet (UF_in_) and at the outlet (UF_out_) of the cartridge at the start or at the end of a 240 min HFR session in 12 inflamed chronic HD patients. The pro-inflammatory activity of the ultrafiltrate samples was also determined by evaluating the changes that they induced in IL-6 mRNA expression and protein release in peripheral blood mononuclear cells from 12 healthy volunteers. IL-6 and p-cresol circulating levels were also assessed in peripheral plasma blood samples collected before and after HFR and, for comparison, a control HD.

**Results:**

p-Cresol and IL-6 were lower in UF_out_ than in UF_in_ both at the start and at the end of the HFR session, suggesting that they were retained by the cartridge. IL-6 mRNA expression and release were lower in PBMC incubated with UF_out_ collected at the end than with UF_in_ collected at the start of HFR, suggesting that passage through the cartridge reduced UF pro-inflammatory activity. Plasma total p-cresol decreased by about 53% after HFR, and 37% after HD. IL-6 circulating values were unmodified by either these dialysis procedures.

**Conclusions:**

This study shows that the HFR-Supra cartridge retains total p-cresol and IL-6 in the ultrafiltrate and lowers plasma total p cresol but not IL-6 levels.

**Trial Registration:**

ClinicalTrials.gov NCT01865773

## Introduction

Cardiovascular disease (CVD) is the primary cause of morbidity and mortality in End Stage Renal Disease (ESRD): approximately 50% of ESRD patients die because of CVD, and cardiovascular mortality is 15–30 times higher in ESRD than in age-adjusted general population [1], [2]. This excess in morbidity and mortality cannot be explained only by the high prevalence of the traditional Framingham risk factors in these patients [3], [4]. It seems, instead, to be also dependent on Chronic Kidney Disease (CKD)-specific, non-classical factors, such as inflammation [5], vascular calcification [6], anemia and left ventricular hypertrophy [7]. A wealth of data points to the retention of organic waste solutes as one of the mechanisms leading to the development of these non-traditional risk factors in CKD. In particular, a major role has been recently attributed to p-cresol and to its metabolites. This aromatic compound is generated in the gut through the bacterial degradation of tyrosine and phenylalanine [8], [9]. Once absorbed from the intestinal mucosa, p-cresol is almost completely converted in two main metabolites, p-cresylsulfate and p-cresylglucuronide that are efficiently removed by the kidney [10]. Observational studies showed that the metabolites of p-cresol accumulate in the plasma of patients affected with CKD. It was also shown that high plasma concentrations of total p-cresol and, specifically, of its p-cresylsulfate metabolite, are independently associated with CVD and mortality, even after adjustment for known risk factors [10]–[12]. Available data suggest that p-cresylsulfate may cause a diffuse endothelial damage [13]–[15] and increase the percentage of leucocytes displaying oxidative burst activity [16]. Therefore a role has been posited for p-cresol and its metabolites as a factor contributing to the systemic inflammation that is commonly observed in dialysis patients [17], [18].

In ESRD, there is also a strong association between elevated circulating levels of markers of inflammations such as C-reactive protein (CRP) and interleukin-6 (IL-6) and cardiovascular morbidity and mortality, suggesting that systemic inflammation could have a pathogenic role in CVD in these patients [19]–[23]. This could in part be due to the ability of circulating cytokines, like IL-6, to exert a direct pro-aterogenic effect or to regulate the release of acute phase reactants [24].

Combined, these data suggest that cardiovascular prognosis in ESRD patients could be improved by decreasing the circulating concentration of p-cresol and its metabolites and/or of IL-6. Unfortunately, both conventional bicarbonate hemodialysis (HD) and hemodiafiltration (HDF) may remove p-cresol only with a low efficiency and they are unable to clear the plasma from IL-6 [25]–[27]. Indeed, although the molecular weight of p-cresol and its metabolites is very low, ranging around 100 Da, these compounds cannot cross the dialysis membrane because they circulate in plasma mostly bound to albumin being their free percentage less than 1% [28]. Conversely, IL-6, also in its free form (MW 24.5 kDa), is a large, difficult to dialyze solute [29]. Moreover, only about 30% of circulating IL-6 is free and the remaining 70% circulates bound to its two soluble receptors, sIL-6R (MW 55 kDa) and sgp80 (100 kDa), forming very high molecular weight complexes [30].

Alternative dialysis strategies have been developed with the aim of improving the efficiency of protein-bound compounds removal from plasma. They are based either on the adsorption of these compounds on resin cartridges [31], [32], or on strategies that could lower the binding of uremic toxins to plasma proteins [33]. One of the most promising among the former is Hemo-Filtrate-Reinfusion (HFR), a new dialysis technique that combines the processes of diffusion, convection and adsorption [34]. In this double chamber hemodiafiltration system, the UF is reinfused into the blood of the patient after its passage through a resin cartridge that binds many medium-high molecular weight solutes including some pro-inflammatory cytokines [35]. No data are available on the ability of this dialysis system to clear the blood of IL-6 and p-cresol or its metabolites. Therefore, we designed the present study to evaluate whether, during a single dialysis session, the HFR sorbent cartridge may efficiently bind and remove from the UF either p-cresol and its metabolites or IL-6 or both of them. To address this question we measured total p-cresol and IL-6 concentrations in the UF produced in the first filter of HFR system, before and after its passage through the sorbent cartridge. We also evaluated whether the pro-inflammatory activity exerted by the UF on peripheral blood mononuclear cells (PBMC), harvested from healthy subjects and cultured *in vitro*, was lowered by the passage through the sorbent cartridge.

## Patients and Methods

### Patient selection and study protocol

This study was performed in 2011 in the Dialysis Unit of Federico II University of Naples. We obtained approval by our ethic committee “Carlo Romano” of the Federico II University and written informed consent from all subjects, according to the Helsinki declaration as revised in 1996. The protocol for this trial and supporting TREND checklist are available as supporting information; see Checklist S1 and Protocol S1.

Inclusion criteria were: the diagnosis of ESRD, the inclusion in a regular three times weekly hemodialysis program and the evidence of ongoing systemic inflammation, as stated by high-sensitivity CRP (hsCRP) concentrations higher than 3 mg/L. We chose to perform the study in patients with high circulating values of hsCRP because they are expected to also have high IL-6 concentrations both in plasma and in the UF.

Exclusion criteria were: malignancies, systemic autoimmune and/or infectious diseases, severe malnutrition or conditions making necessary to artificially feed the patient.

Twelve patients (6 male/6 female) were recruited for the study. Their mean age was 60.1±11.5 years; mean dialysis vintage was 80±39 months. Plasma high-sensitivity CRP was above the upper normal limit in all patients (mean ± SD values: 44.01±22.18 mg/l, normal values <3.0 mg/l).

The primary endpoint of the study was to establish whether IL-6 and p-cresol were retained on the HFR cartridge during a single dialysis session. To assess this point, we compared the concentrations of these uremic toxins in UF entering (UF_in_) and UF exiting (UF_out_) from the cartridge, collected 15 minutes after the start (start-15 min) and 15 minutes before the end (end-225 min) of the HFR session.

Secondary endpoints were: 1) to assess whether the pro-inflammatory activity of the ultrafiltrate (UF) was lowered after the passage through the HFR cartridge and 2) whether a single HFR session decreased the circulating concentrations of total p-cresol and IL-6 more efficiently than HD. To address the first point, we compared the ability of UF samples collected at the beginning and at the end of the HFR session to induce an inflammatory response in peripheral blood mononuclear cells (PBMCs) drawn from healthy subjects in vitro, as indicated by an increase in IL-6 gene expression and release. To establish whether HFR is more effective than HD in lowering IL-6 and p-cresol serum concentrations, we compared the circulating levels of these uremic toxins in blood samples collected before and after a single session of either HFR or HD performed at different times in the same patients.

### HFR treatment

**Figure 1:**
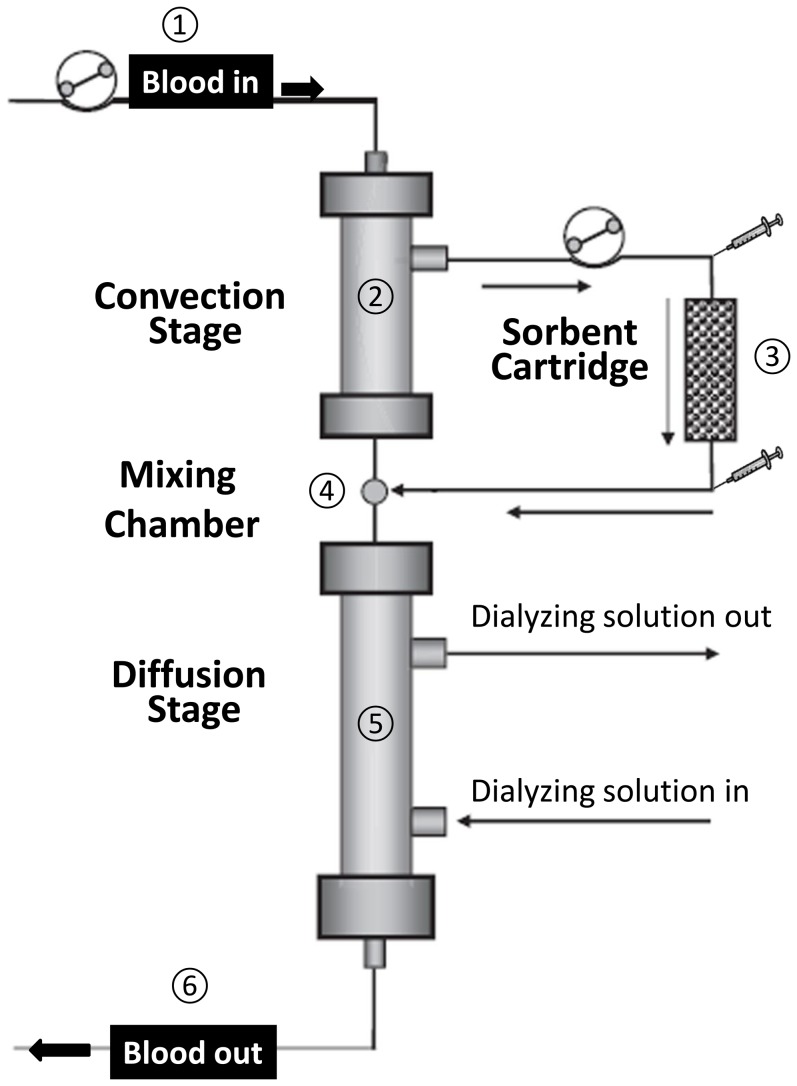
Schematic representation of the HFR apparatus. The figure shows the main components of the HFR Supra system (Bellco, Mirandola, Italy) used in the study. 1-The blood of the patient is pumped into the top filter (the convection stage). 2-In this stage of the HFR apparatus, the blood is filtered by a high flux polyethersulfone membrane. 3-The ultrafiltrate produced in the convection stage is pumped by a second pump through a sorbent cartridge where hydrophobic waste solutes are retained. The small syringes indicate the points of the systems where rubber stopper for UF sample collection are located. 4-The blood coming from the convection stage and the UF coming from the cartridge are mixed in a chamber located between the two filters 5-This “reconstituted” blood enters the bottom filter (the diffusion stage) where it is undergoes dialytic treatment (both diffusion and ultrafiltration for weight loss) with a low flux polyethersulfone membrane. 6-The cleared blood is returned to the patient. (Modified form an original image kindly provided by Dr. G. Palladino, Bellco, Mirandola, Italy).

HFR was performed using a commercial HFR Supra Bellco filter system (Mirandola, Italy). This system consists of a convective stage, of a sorbent cartridge and of a diffusive stage (Fig. 1). Therefore, in the HFR apparatus, the processes of convection, diffusion and adsorption are physically separated. During HFR, the blood first goes through the 0.7 m^2^ high-flux polyethersulfone (polyphenylene) membrane of the convective stage. This highly biocompatible membrane [36] has a sieving coefficient (i.e. the ratio between solute concentrations in ultrafiltrate and plasma) for albumin of 0.02 and, therefore, it generates an ultrafiltrate (UF) containing small amounts of albumin. The UF generated in the convective stage then goes through a sorbent cartridge containing 40 ml of a hydrophobic styrene resin (Selecta; Bellco, Mirandola, Italy) that, because of its high porosity, has a very large adsorption surface area (∼700 m^2^/g). The cartridge adsorbs by hydrophobic interaction a number of waste solutes contained in the UF that is, thus, cleaned. Thereafter, this “regenerated” UF is reinfused into the blood stream being mixed with the blood coming from the convection stage into a chamber located at the bottom of the convection stage before the diffusive section of the filter. The second filter, the diffusive stage of the HFR apparatus, contains a 1.7 m^2^ low-flux polyethersulfone (polyphenylene) membrane (Figure 1). It mostly operates by diffusion and is also responsible for the removal of excess water and patient's weight loss.

The HFR dialysis system was controlled by a Formula Plus monitor (Bellco, Mirandola, Italy) equipped with a software that automatically determines the best ultrafiltration flow rate (Q_uf_) [37]–[39]. Mean blood and dialysate flows were 330±20 ml/min and 545±85 ml/min, respectively. The Q_uf_ from the convective section and the ultrafiltrate-reinfusion rate were kept at 50–60 ml/min. Mean dialytic time was 240±15 min. Weight loss, that was obtained only in the diffusive section of the system, was 800±100 g/h.

The sterility of the tap water, of the water obtained after reverse osmosis and of the dialysate was periodically checked by colony forming unit (CFU) testing and by performing the limulus amebocyte test (LAL) test (gel clot test) for endotoxin (PBI, Milan, Italy). These tests gave negative results during the entire duration of the study.

### Control hemodialysis

Because both the convective and the diffusive stages of the HFR apparatus can remove waste solutes, they could both contribute to HFR effect on plasma p-cresol or IL-6. Therefore, in order to dissect their respective roles, we also measured the changes of total p-cresol and IL-6 plasma levels after a pure diffusive hemodialysis session performed using only the second stage of the HFR system. This represented an internal control for the HFR experiment and gave an estimate of the HFR cartridge-independent effects. Hemodialysis (HD) was performed with the same monitor (Bellco Formula) and the same low flux (1.7 m^2^) polyethersulfone membrane (Diapes, Bellco, Italy) and the same conditions (dialysate and dialysis parameters including dialysate and blood flow rates, weight loss and dialysis efficiency, as KT/V) of HFR.

### Ultrafiltrate and blood samples

Four 60 ml UF samples were collected through specific rubber stoppers located at the inlet and at the outlet of the sorbent cartridge, using sterile disposable syringes (Fig. 1). Two of these samples were collected 15 minutes after the beginning of the HFR session (15 min-start), the first (UF_in_-15) before and the second (UF_out_-15) after the passage through the adsorbent cartridge. The other two samples were collected 225 min after the beginning of the treatment (i.e. 15 minutes before its end, 225 min-end), also in this case one before and the other after the passage through the cartridge, (UF_in_-225 and UF_out_-225, respectively). Immediately after collection, the UF samples were transferred in sterile disposable polypropylene tubes (BD-Falcon, Milan, Italy), stored in ice and transferred into −80° refrigerator where they were kept until assayed for p-cresol and IL-6 determinations or used for the experiments in vitro with human PBMC, as described below.

In all patients, 10 ml blood samples were collected from A-V fistula 15 minutes before the beginning and 15 min after the end of both the HFR and of the HD session. Sera were separated immediately after centrifugation and stored at −20° until assayed.

Albumin concentrations both in serum and UF samples were measured by nephelometry. p-Cresol and IL-6 serum concentrations were measured using the techniques detailed in the next paragraph, whereas high-sensitivity C-reactive protein (hsCRP) was evaluated by a modified high-sensitivity laser nephelometry technique (Berhing Diagnostics, GmbH, Marburg, Germany).

### p-cresol assay

Total p-cresol (i.e. the sum of its free and protein-bound fractions) was measured in serum and UF samples with HPLC, as previously described [40], [41]. Briefly, after heat-acid denaturation of the binding proteins, p-cresol was extracted in ethyl acetate and injected on the HPLC column. The UF samples (50 ml) were vacuum dried and the obtained pellets were resuspended in 3 ml saline solution before being denaturated and extracted. The preliminary acidification of the samples was performed to release bound p-cresol conjugates from plasma proteins, to make total (bound + free) p-cresol-conjugates available for further HPLC analysis. Plasma acidification also caused the hydrolysis of both p-cresylsulphate and p-cresylglucuronide that were converted in p-cresol. Therefore, this method mainly measured p-cresylsulfate that represents the most prevalent form of p-cresol in plasma [10]. The limits of quantification (LOQ) and of detection (LOD) were 0.150 ng/mL and 0.017 ng/ml, respectively. Calibration procedures were repeated every two weeks (coefficient of variation <0.5%).

### Correction for hemoconcentration

The values of the concentrations of the two protein bound solutes total p-cresol and IL-6 were corrected, in the samples drawn at the end of the treatment, for the hemoconcentration that occurs during dialysis by using the method proposed by Lesaffer et al. with modifications [42]. Briefly, the concentrations measured in blood samples collected at the end of the dialysis session were multiplied by a correction factor (CF) consisting in the ratio between total serum albumin concentration at the beginning (Alb_t0_) and at the end of dialysis (Alb_t240_): CF = Alb_t0_/Alb_t240_.

### IL-6 gene expression and release

To assess the pro-inflammatory activity of the different UF samples, collected as described in the previous section, we evaluated their ability to induce IL-6 gene expression and IL-6 release in cultured PBMCs.

PBMC were isolated from 12 healthy subjects with normal hsCRP concentrations (1.15±0.8 mg/l) by Ficoll-Hypaque (Flow Laboratories, Irvine, UK) gradient density centrifugation (400×g for 30 min). Cell viability, assessed by trypan blue dye exclusion, was higher than 95%. PBMCs were resuspended in RPMI culture medium (Sigma, Milano, Italy) supplemented with 1% heat-inactivated FCS (Sigma) and antibiotics (penicillin and streptomycin) at a density of 1×10^6^/ml, subdivided in three different aliquots in plastic culture tubes (BD-Falcon, Milan, Italy) and cultured for 24 h at 37°C in a 5% CO_2_ saturated humidity incubator after the addition of 20% v/v NaCl (0.9%) to the first aliquot, 20% v/v UF_in-15_ to the second aliquot and 20% v/v UF_out-225_ to the third. At the end of the 24 incubation period, cell-free supernatants were collected by centrifugation and stored at −80°C until assayed for IL-6 levels whereas the cell pellets were lysed with Trizol reagent (Life Technologies, Grand Island, NY) for total RNA extraction, as described elsewhere [30]). The concentration and the purity of RNA were evaluated by measuring the absorbance at 260 nm and by calculating the ratio of absorbance at 260–280 nm using a UV spectrophotometer (DU-800 spectrophotometer; Beckman Instruments). Five micrograms of total RNA were retro-transcribed and Real-Time quantitative PCR analysis was performed with an ABI prism 7500 (Applied Biosystems, Foster City, CA) apparatus and the TaqMan system using primers specific for IL-6 mRNA and β-actin (sequence reported in Table 1). IL-6 mRNA levels were normalized to those of β-actin and converted into fold changes based on the doubling of PCR product in each PCR cycle according to the manufacturer's guidelines, as previously described [43].

**Table 1:** Sequence of the IL-6 and β-actin primers used in the study.

	Forward	Reverse	Reference
**IL-6**	ATG AAC TCC TTC TCC ACA AGC GC	GAA GAG CCC TCA GGC TGG ACT G	30
**β-actin**	CACCATGGATGATGATATCG	TGGATAGCAACGTACATGG	51

### Statistical analysis

Data are expressed as mean ± standard deviation. They were analyzed using SPSS 12.0 (SPSS Inc, Chicago, IL) setting the threshold for statistical significance at p values <0.05. The Shapiro-Wilk test was performed to assess whether the different sets of data were normally distributed or not. To evaluate percent decrease of IL-6 and total p-cresol plasma concentrations we calculated their *reduction ratios* according to the formula RR = (C_before_-C_after_)/C_before_, where C_before_ and C_after_ indicate the sera concentrations in blood samples collected 15 min before and 15 after either the HFR or the HD session. Statistical comparisons of two group variables were performed using Student's t-test for normally distributed data and Mann-Whitney test for not-normally distributed data. Multiple group comparisons were performed with one way repeated measure ANOVA followed by the Student-Newman-Keuls post hoc test.

## Results

### IL-6, p-cresol and albumin concentrations in UF samples

We found measurable concentrations of both total p-cresol and IL-6 in UF_in_-15 that is produced by the ultrafiltration of plasma through the polyethersulfone membrane of the first stage convection filter. In UF_in_-15, the concentration of p-cresol was about 30 times lower (0.39±0.2 vs 11.6±6.3 mg/l) and that of IL-6 was 5.6 times lower than in plasma (10.30±10.2 vs 57.6±58.3 pg/ml) (Tables 2 & 3). To assess whether a significant amount of albumin crossed the polyethersulfone membrane of the convection filter, we measured this protein in the UF_in_-15. The concentration that we found ranged around 1/40^th^ of plasma values (0.1±0.03 vs 3.6±0.2 g/dl) in agreement with the value of 0.02 of albumin sieving reported by the manufacturer of these dialysis membranes. Intriguingly, these albumin concentration values are consistent with the hypothesis that most of the filtered p-cresol actually crosses the dialysis membrane as protein-bound forms. Conversely, the percent values of IL-6 concentration was close to that of free IL-6 in plasma suggesting that only free IL-6 could cross the dialysis membrane.

**Table 2:** Albumin, total-cresol and IL-6 concentrations in ultrafiltrate samples collected before and after the HFR sorbent cartridge at the beginning and at the end of the dialysis session.

	15 min Pre-cartridge	15 min Post-cartridge	225 min Pre-cartridge	225 min Post-cartridge
**Albumin (g/dl)**	0.1±0.03	0.08±0.02*	0.09±0.01	0.07±0.01^†^
**Total-Cresol (mg/l)**	0.39±0.2	0.14±0.09*	0.25±0.09*	0.04±0.03*
**IL-6 (pg/ml)**	10.30±10.2	0*	11.68±11.15	0.91±1.01*

The data shown in the Table are the mean ± standard deviation of albumin, total cresol and IL-6 measured in samples of the UF collected before and after being adsorbed by the sorbent cartridge of the convection stage of the HFR Supra apparatus. Note the very low concentrations of albumin in pre-sorbent UF samples that is consistent with the low sieving coefficient (about 0.02 for albumin) of the polyphenylene membrane of the system. The values of total cresol concentration shown are the sum of p-cresol and p-cresol metabolite concentrations because, as we detail in the methods section, the acidic extraction method that we used converts all p-cresol derivatives in p-cresol.

*p<0.05 vs 15 min pre-cartridge and ^†^p<0.05 vs 225 min pre-cartridge, at repeated measure ANOVA followed by the Student-Newman-Keuls post-hoc test.

**Table 3:** Serum albumin, and plasma total cresol and Il-6 concentrations before and after either HFR or HD.

	Before HFR	After HFR	Reduction Ratio (%)	Before HD	After HD	Reduction Ratio (%)
**Albumin (g/dl)**	3.6±0.2	4.2±0.3	-	3.8±0.3	4.2±0.2	-
**Total Cresol (mg/l)**	11.6±6.3	5.8±2.7*	53.6±12.5	8.6±5.3	5.4±3.0∥	37.1±20.2°
**IL-6 (pg/ml)**	57.6±58.3	48.4±53.1	4.3±34.5	48.7±33.6	53.9±48.3	-

The Table reports the mean ± standard deviation of the values of serum albumin, plasma total cresol and IL-6 concentrations obtained in the same 12 ESRD patients before and after two different dialysis sessions performed with standard bicarbonate hemodialysis (HD) or with HFR supra, as indicated. The Table also reports the Reduction Ratio of p-cresol and IL-6 obtained with both HFR and HD. As described in the legend to Table 2, total cresol concentration is the sum of the concentration of p-cresol and of its metabolites. The post-dialysis data shown in the table have been corrected for hemoconcentration as reported in the Methods section.

*p<0.005 vs preHFR,

∥ p<0.005 vs preHD at Mann-Whitney test;

° p<0.05 vs RR HFR at Student's t test for paired data.

To establish whether p-cresol and IL-6 in the UF_in_ could be adsorbed by the sorbent cartridge of the HFR system we compared the concentrations of these uremic toxins in UF_in_ and UF_out_. As reported in Table 2, in samples collected 15 min after the beginning of the HFR session both total p-cresol and IL-6 concentrations were significantly lower in UF_out_ than in UF_in_. Also albumin concentration was lower in UF_out_ than in UF_in_ (Table 2). These findings suggested that the sorbent cartridge could retain p-cresol, IL-6 and a small amount of albumin. To assess whether the ability of the cartridge to adsorb these substances was maintained till the end of the HFR session, we also measured their concentrations in UF_in_-225 and UF_out_-225. Total cresol concentration in UF_in_-225 was lower than in UF_in_-15, and it was further lowered by the passage through the cartridge, suggesting that the binding capacity of the cartridge was not saturated. In keeping with this hypothesis, albumin concentration in UF_in_-225 was also significantly lowered upon passage through the cartridge. Also IL-6 concentration was significantly higher in UF_in_-225 than in UF_out_-225. However, the concentration of this cytokine in UF_in_-225 was not different from that in UF_in_-15 (Table 2).

### Effect of the passage through the sorbent cartridge on IL-6 mRNA expression and protein release induced by the ultrafiltrate in PBMC from healthy subjects

**Figure 2:**
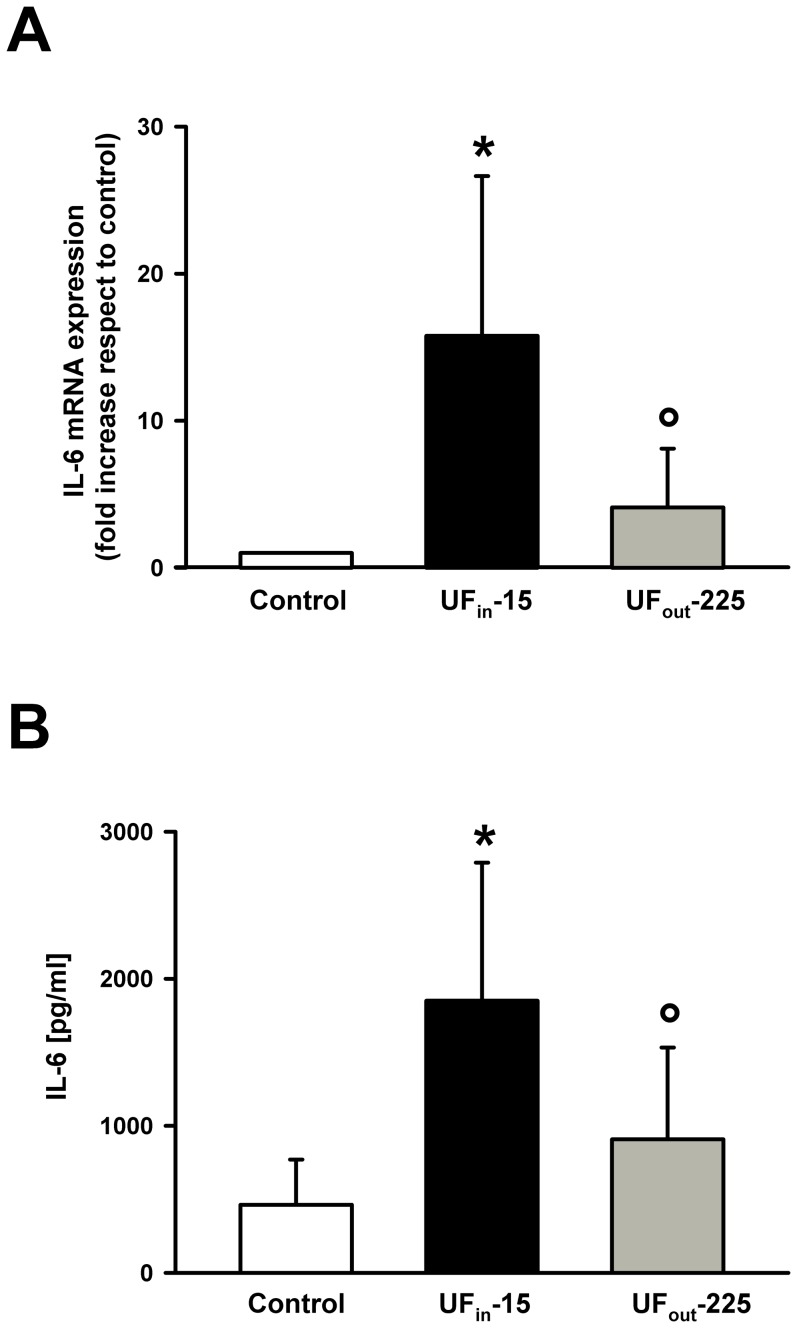
Effect of UF_in_-15 and UF_out_-225 on IL-6 gene expression and on IL-6 release in PBMC cultures from healthy subjects. A Mean ± SD of the fold increase in IL-6 gene expression respect to controls observed in PBMC cultures from 12 healthy volunteers, exposed to UF_in_-15 (black) and UF_out_-225 (dark gray). Data were normalized to β-actin mRNA expression and expressed as percent increase respect to those obtained in control cultures, set at 1, in which no UF was added. B, Mean ± SD of immunoreactive IL-6 concentrations in culture media of PBMCs from twelve healthy volunteers after a 24 h incubation with saline (basal condition, white), UF_in_-15 (black), or and UF_out_-225 (dark gray). *p<0.05vs control; °p<0.05 vscontrol and UF_in_-15, at Kruskal-Wallis one way analysis of variance on ranks followed by Student-Newman-Keuls post hoc test.

To establish whether the passage through the HFR cartridge could also decrease UF pro-inflammatory activity, we compared IL-6 gene expression in PBMC from healthy subjects cultured in the absence (Control) or in the presence of the UF collected either at the beginning (UF_in_-15) or at the end of the HFR session (UF_out_-225). In addition, we compared IL-6 concentrations in the culture media obtained by the same PMBC cultures. As expected, in the presence of UF_in_-15 normal PBMCs became activated and we observed a significant increase respect to control cultures both in IL-6 mRNA expression (15.76±10.9 fold the basal expression, p<0.02) and IL-6 concentrations in the culture medium (1850.92±938.43 vs 462.96±307.28 pg/ml, p<0.01) (Fig. 2). In PBMCs exposed to UF_out_-225 IL-6 mRNA levels were significantly higher than in control cultures but significantly lower than in cultures treated with UF_in_-15 (4.10±3.99 fold the basal expression, p<0.05). Similarly, IL-6 release was higher than in controls but lower than in PBMCs incubated withUF_in_-15 (462.96±307.28 vs 908.5±623.59 pg/ml p<0.05) (Fig. 2).

### Effect of HFR and HD on plasma of IL-6 and total p-cresol

To assess whether the HFR session could also modify the circulating levels of either total p-cresol or IL-6, we compared the plasma concentrations of these uremic toxins in blood samples collected from the AV fistula 15 min before the beginning and 15 min after the end of the HFR. Because a significant hemoconcentration occurred during the dialysis, as expected [39] and as indicated by the higher albumin concentration at the end of HFR than at its beginning (Table 3), total p-cresol and IL-6 concentrations were corrected for this factor (see the methods section for details). Total p-cresol plasma concentration was significantly lower after HFR than before, whereas IL-6 level did not change (Table 2). To evaluate whether HFR was more effective than HD in lowering total p-cresol plasma concentrations, we also measured the concentrations of this toxin in blood samples collected in the same patients before and after a standard HD session. Total p-cresol reduction ratio was significantly higher after HFR than after HD (53.6±12.5% vs 37.1±20.2%, n = 12; p<0.05 at Student's t-test for paired data) (Table 3). Conversely, even after HD, IL-6 concentrations were not significantly modified by the treatment (Table 2).

## Discussion

Because uremic protein-bound toxins are suspected to have a role in worsening the cardiovascular prognosis in patients on dialysis, there is a growing interest in developing therapeutic strategies more effective than those currently available in removing these compounds. In the present study we explored whether the HFR Supra dialysis system could have a role in this context. Specifically, we reported evidence that its sorbent cartridge removed both IL-6 and p-cresol and its metabolites from the UF and significantly lowered the UF pro-inflammatory activity in vitro. Parallel to these effects, we observed a significantly stronger decrease in circulating levels of total cresol, but not of IL-6, after HFR than after HD.

Schematically the HFR apparatus may be considered as composed by two different systems both taking part to waste removal. The first one is a convective stage that operates an ultrafiltration process and is coupled with a sorbent cartridge. The second is a diffusive stage essentially similar to conventional HD. As far as the first stage of HFR is concerned, the only mechanism that contributes to the removal of toxins and waste compounds is binding and retention on the HFR cartridge. The UF generated by blood ultrafiltration at this HFR stage is, indeed, entirely reinfused in patient's general circulation after being passed through the sorbent cartridge. Therefore, all the compounds that are not sequestered into the cartridge are actually returned to patient's blood. The results of our study are consistent with the hypothesis that both IL-6 and p-cresol and its derivatives were actually adsorbed by the HFR cartridge in our patients. This is mainly indicated by the evidence that the concentrations in the UF of these uremic toxins were significantly lowered after the passage through the cartridge. The hypothesis that the sorbent cartridge could bind and retain IL-6 and/or p-cresol and its metabolites is consistent with the chemical-physical characteristics of its styrene resin that can establish strong non-covalent interactions with hydrophobic molecules and with albumin [35].

A relevant question arises about the forms in which either p-cresol or IL-6 bind to the resin. Both these molecules circulate, indeed, in the plasma both as protein-bound and as free forms. Specifically, p-Cresol and its derivatives are more than 90% bound to albumin whereas almost 70% of IL-6 circulates as a very high molecular complex with its soluble receptors (sIL-6R and sgp130) [30], [44]. The filtering membrane of the convection stage of the HFR Supra apparatus (Fig. 1) has a low but measurable permeability to albumin with an albumin sieving coefficient of 0.02. Therefore, it lets an amount of albumin ranging around 1/40^th^ of its plasma concentration cross the membrane and appear into the UF. This implies that uremic toxins, like p-cresol, can be transferred in the UF also in their protein bound forms. In addition, the free forms of p-cresol and of its metabolites are small enough to freely permeate through the convection stage filter. Therefore, both protein-bound and free p-cresol presumably passed through the dialysis membrane into the UF and become available for binding to the cartridge. Conversely, because of the very high molecular weight of its complex with soluble IL-6 receptors, it is likely that IL-6 passed into the UF only as a free molecule. This is consistent with the evidence that IL-6 concentration in the UF averaged 1/5^th^ of the plasma concentration, the value expected for free IL-6 (MW 24.5 kDa) crossing through a membrane with IL-6 sieving coefficient of about 0.3. The evidence that we found low or undetectable IL-6 concentrations in the effluent from the cartridge suggests that the IL-6 that crossed the dialysis membrane of the first HFR stage to move into the UF was virtually all retained by the cartridge itself. In keeping with this result is the evidence that the ability of the UF incubation to trigger IL-6 gene expression and release in PBMC from healthy subjects was greatly reduced after its passage through the HFR cartridge sorbent bed.

While the evidence discussed above suggests that HFR cartridge contributed to clear the plasma of our patients from uremic toxins, a role could have been played also by the second, diffusive stage of the HFR apparatus [26]. As mentioned above, this portion of the HFR system operates as a conventional HD system in which diffusion represents the main mechanism of solute removal. However, the removal of p-cresol by this mechanism is expected to be small (regarding only its free form) and that of IL-6 null. Indeed, less than 1% of total p-cresol circulates as free form whereas free IL-6 is still too large to cross the low flux membrane of the second HFR stage. As a whole these considerations suggest that a significant amount of p-cresol and all of IL-6 removal actually took place in the first HFR stage. This can probably account for the significantly higher decrease of plasma p-cresol level that we observed in our patients when they underwent HFR as compared to HD (53.6% vs 37.1%). Interestingly, similar values of percent decrease in plasma p-cresol concentration after HD were recently reported by Meert et al. [45]. Therefore, our data suggest that HFR could perform better than HD in p-cresol removal though this conclusion remains to be confirmed in larger studies specifically designed to address this point. Finally, regarding classical hemodiafiltration (HDF), a previous work showed that total p-cresol concentration decreases by 40% after post-dilution and 42% after pre-dilution HDF [46] but no comparative study with HFR has ever been performed.

Although the HFR cartridge retained IL-6, we did not observe any significant change in serum concentrations of this cytokine when we compared blood samples collected before and following a single HFR session. This finding could be explained considering that the actual amount of IL-6 removed during a single HFR is presumably small. Based on concentration in UF and on the value of Q_uf_ in the HFR system, we estimated that less than 10% of circulating IL-6 could be removed by the cartridge. Remarkably, no additional IL-6 removal can occur in the diffusive stage of the HFR apparatus because free IL-6 cannot be dialyzed by the low flux membrane of its filter. It is likely that the small amount of IL-6 removed by HFR could be entirely replaced either by the new synthesis of this cytokine or by its release from tissues. It has been suggested, indeed, that in inflamed patients, circulating IL-6 remains high despite its very short plasma half-life because it is produced at a very high rate [27]. Under this respect we should consider that all our patients showed a remarkable systemic inflammation as demonstrated by the high serum concentrations of hsCRP. It should be emphasized at this point, however, that such a marked systemic inflammation is not the most typical finding in ESRD patients that, instead, usually present only a moderate inflammatory state known as “microinflammation” [47]. We can speculate, thus, that HFR that was ineffective against the high IL-6 concentration of our markedly inflamed patients could perform better in the average ESRD patient with microinflammation.

Our findings suggest that HFR does not differ from other dialysis systems that have all been shown ineffective in lowering the circulating levels of IL-6 and/or of other cytokines. Specifically, IL-6 levels did not change after a single HD session, as shown by Tarakçioğlu et al. [27] and as also observed by us in the present paper. In addition, continuous hemofiltration was also ineffective in lowering circulating IL-6 levels in patients with systemic inflammatory response syndrome [48]. After measuring the very small amounts of several cytokines such as TNFα and IL-1 that are removed by low-volume HF (Vuf approximately 12 L/day), Van Bommel et al. [49] estimated that a UF volumes of at least 50 L/day should be needed to clear the plasma from these molecules using hemofiltration.

While a single HFR session seems to be ineffective in decreasing plasma IL-6, a substantial lowering of the circulating level of this cytokine has been reported with repeated HFR treatment. Specifically, Panichi et al. [29] showed that IL-6 plasma concentration significantly decreased respect to baseline after 4 months of repeated HFR treatment. This evidence suggests that longer follow up of HFR treatments could slowly decrease plasma IL-6 levels by inducing a decrease in systemic inflammation. A significant decrease of oxidative stress was also reported by Calò et al in patients receiving HFR [50].

In conclusion, our data provided the proof of concept that the HFR system could represent a new method to remove p-cresol and IL-6 from the blood. It would be interesting to evaluate whether HFR efficiency could be increased by the new generation of membranes with a tenfold higher sieving coefficient for albumin (0.2) that are becoming available. These new HFR systems are expected to greatly increase the amount of albumin bound p-cresol and, presumably, of other inflammatory mediators that can be retained by the cartridge and, therefore, to further increase their advantages respect to HD in protein bound solutes removal.

## Supporting Information

Checklist S1 **TREND Checklist.**
(DOCX)

Protocol S1 **Trial Protocol.**
(DOCX)
