# Supplementary material for: Evidence That p-Cresol and IL-6 Are Adsorbed by the HFR Cartridge: Towards a New Strategy to Decrease Systemic Inflammation in Dialyzed Patients?
Source: PLoS One. 2014 Apr 22;9(4):e95811. doi: 10.1371/journal.pone.0095811 (PMC3995921; doi:10.1371/journal.pone.0095811)
Supplement: Protocol S1 — Trial Protocol. (DOCX) [file pone.0095811.s002.docx]

**STUDY PROTOCOL**

| **Official Title** | Role of HFR Cartridge in the Removal of Mediators of Inflammation and P-cresol in Hemodialysis Patients |
| --- | --- |
| **Short Title** | HFR Cartridge and Inflammation |
| **Start Date** | January 2011 |
| **Brief Summary** | A major limitation of standard hemodialysis is that it does not clear the plasma from interleukin-6 (IL-6) and p-cresol, two uremic toxins responsible for the high cardiovascular risk in end stage renal disease (ESRD). In the present study, we evaluated whether these compounds are removed by HFR-Supra, a double-chamber hemodiafiltration system in which the ultrafiltrate (UF) returns to the patient after its regeneration through a resin cartridge. We selected 12 inflamed chronic hemodialysis (HD) patients, which underwent a single 240 minutes HFR session. We studied the change in both IL-6 and p-cresol circulating levels, by comparing pre- and post-HFR serum concentrations. In addition, we compared Il-6 and p-cresol levels in the UF entering (UFin) and exiting (UFout) from the cartridge, either at the start or at the end of the HFR session. The proinflammatory activity of UFin and UFout was determined by evaluating the changes that they induced in IL-6 messenger ribonucleic acid (mRNA) expression and release in peripheral blood mononuclear cells (PBMC) collected from 8 healthy volunteers and cultured in vitro for 24 hr. |
| **Study Design** | Intervention Model: Single Group Assignment Masking: Open Label |
| **Condition** | Chronic hemodialysis |
| **Intervention** | Procedures: HFR dialysis and control hemodialysis (using the same membrane) performed in the same subjects receiving HFR.  HFR is a dialysis technique that combines the processes of diffusion, convection and adsorption. In this double chamber hemodiafiltration system, the ultrafiltrate is reinfused after its passage (and opportune modification) through a resin cartridge.  Other Name: hemodiafiltration with on line reinfusion of ultrafiltrate. |
| **Study Arm (s)** | Single arm.: Experimental: HFR  We selected 12  inflamed chronic HD patients, which underwent a single 240 minutes HFR session  Intervention: Procedure: HFR dialysis |
| **Eligibility Criteria** | Inclusion criteria were: the diagnosis of ESRD, the inclusion in a regular three times weekly hemodialysis program and the evidence of ongoing systemic inflammation, as stated by high-sensitivity CRP (hsCRP) concentrations higher than 3 mg/L. We chose to perform the study in patients with high circulating values of hsCRP because they are expected to also have high IL-6 concentrations both in plasma and in the UF.  Exclusion criteria were: malignancies, systemic autoimmune and/or infectious diseases, severe malnutrition or conditions making necessary to artificially feed the patient. |
| **Gender** | Both males and females |
| **Ages** | 18 Years to 70 Years |
| **Accepts Healthy Volunteers** | Yes, to provide pBMCs |
| **Primary Endpoints** | To establish whether IL-6 and p-cresol were retained on the HFR cartridge during a single dialysis session |
| **Primary Endpoint Measure** | - IL-6 removal by a single session of HFR-Supra [Time Frame: The evaluation will be performed for the duration of a single HFR session of 240 minutes ] [ Designated as safety issue: No ]   We studied the change in IL-6 circulating levels, by comparing pre- and post-HFR serum concentrations. In addition, we compared IL-6 levels in the UF entering (UFin) and exiting (UFout) from the cartridge, either at the start or at the end of the HFR session.   - P-cresol removal by a single HFR session [ Time Frame: The evaluation will be performed for the duration of a single HFR session of 240 minutes ] [ Designated as safety issue: No ]   We studied the change in p-cresol circulating levels, by comparing pre- and post-HFR serum concentrations. In addition, we compared p-cresol levels in the UF entering (UFin) and exiting (UFout) from the cartridge, either at the start or at the end of the HFR session. |
| **Secondary Endpoints** | - 1) to assess whether the pro-inflammatory activity of the ultrafiltrate (UF) was lowered after the passage through the HFR cartridge; - 2) whether a single HFR session decreased the circulating concentrations of total p-cresol and IL-6 more efficiently than HD. |
| **Secondary Endpoint Measure** | - To assess whether the pro-inflammatory activity of the ultrafiltrate (UF) was lowered after the passage through the HFR cartridge, we compared the ability of UF samples collected at the beginning and at the end of the HFR session to induce an inflammatory response in peripheral blood mononuclear cells (PBMCs) drawn from healthy subjects in vitro, as indicated by an increase in IL-6 gene expression and release. - To establish whether HFR is more effective than HD in lowering IL-6 and p-cresol serum concentrations, we compared the circulating levels of these uremic toxins in blood samples collected before and after a single session of either HFR or HD performed at different times in the same patients. |
